# Psychometric Testing of the Chinese Version of the Coping and Adaptation Processing Scale-Short Form in Adults With Chronic Illness

**DOI:** 10.3389/fpsyg.2020.01642

**Published:** 2020-07-23

**Authors:** Xiyi Wang, Leiwen Tang, Doris Howell, Jing Shao, Ruolin Qiu, Qi Zhang, Zhihong Ye

**Affiliations:** ^1^Department of Nursing, Zhejiang University School of Medicine Sir Run Run Shaw Hospital, Hangzhou, China; ^2^Lawrence S. Bloomberg Faculty of Nursing, University of Toronto, Toronto, ON, Canada; ^3^Department of Nursing, Zhejiang University School of Medicine, Hangzhou, China

**Keywords:** psychometrics, coping, adaptation, chronic illness, instrument, Chinese translation

## Abstract

**Background:**

Adaptive capacity may serve as an indicator of the individuals’ coping behaviors toward illness management and may contribute to day-to-day living with chronic illness and improved quality of life. Practical and well-constructed instruments for measuring adaptation have not been adequately explored. An English 15-item Coping and Adaptation Processing–Short Form (CAPS-SF) for assessing adaptation has been created and validated in line with the underlying tenets of Coping and Adaptation Processing theory, but there is no applicable Chinese version.

**Methods:**

The CAPS-SF was translated and culturally adapted into simplified Chinese. Among Chinese adults with chronic illness, 81 patients were selected for cultural adaptation and 288 patients were approached for psychometric testing. Content validity was evaluated by an expert panel. Construct validity was tested by confirmatory factor analysis. Concurrent validity and predictive validity were analyzed by Spearman correlation coefficient. Reliability was assessed by internal consistency and test–retest coefficients. Floor/ceiling effect was calculated.

**Results:**

Adequate content validity was ensured by the expert panel. A four-factor structure (resourceful and focused, self-initiated and knowing-based, physical and fixed, and positive and systematic) describing individuals’ coping strategies was identified and verified. Concurrent validity and predictive validity were demonstrated by strong correlations with the confrontation of coping mode (*r* = 0.46) and a quality-of-life measure (*r* = 0.58). The McDonald’s omega coefficient of total scale was 0.82. Split-half reliability and test–retest reliability were 0.87 and 0.87. No floor/ceiling effect was present.

**Conclusion:**

The Chinese version CAPS-SF is a theoretically based and culturally acceptable instrument with sound psychometric properties. Further studies are advocated to refine its four-factor structure.

## Introduction

Chronic illness is of long duration and generally slow progression (Corbin and Strauss, 1991; Ambrosio et al., 2015). Although the definitions of chronic disease are not unified, they share common features such as “illnesses that are prolonged, have complex causality, do not resolve spontaneously, are associated with functional impairments, and are rarely cured completely” (Bernell and Howard, 2016). Chronic diseases are globally prevalent and associated with high levels of morbidity and disability that contribute to societal and economic burden (Roth et al., 2018). Because of advancements in health care and increased life expectancy, many individuals will be living with one or more chronic diseases (Suls et al., 2019). A positive human response to chronic health conditions can be characterized by the term adaptation, which has been well documented in the literature (Beutel, 1985; Pollock, 1986; De Ridder et al., 2008; Barone and Waters, 2012). Adaptation and coping strategies are shown to be related and crucial for health management (Lazarus, 1993; Livneh, 1999; Audulv et al., 2016). The challenges of living with chronic illness require an individual to find new ways of coping to adapt to their altered health state. Evidence suggests that coping mechanisms are crucial for health management. Coping strategies are beneficial for the mitigation of disabilities, the improvement of health outcomes, and the enhancement of the quality of life (De Ridder et al., 2008; Ambrosio et al., 2015; Livneh, 2015; Cheng et al., 2019) thereby facilitating adaptation to illness. However, instruments with sufficient reliability and validity for measurement of coping are still required in clinical care and research.

Coping refers to how patients identify and act on the opportunities to handle a new situation (Feifel et al., 1987; De Ridder et al., 2008). Among the coping-related measurement scales, the Ways of Coping Questionnaire (WCQ) developed by Lazarus has been widely used in coping researches (Parker et al., 1993). Previous studies showed that the construct validity of the WCQ could be improved (Parker et al., 1993; Edwards and O’Neill, 1998). The Medical Coping Modes Questionnaire (MCMQ) for assessing coping responses has also been explored in the healthcare field (Feifel et al., 1987). A Chinese version of the MCMQ has been developed for use in clinical settings (Shen and Jiang, 2000). These coping scales identify coping strategies used by individuals facing stress, but their theoretical basis could be enhanced in both cognitive and behavioral domains. Coping with stress has been described as both a process and an outcome in the concept of adaptation (Londono and McMillan, 2015; Roy et al., 2016). Within the perspectives of coping efficacy and adaptation, some scales have been published. The Adaptive Capacity Index based on the theory of general adaptation syndrome was developed to measure the adaptive capacity related to fatigue in patients with cancer (Olson et al., 2011). Although this scale included physiological, emotional, cognitive, and behavioral aspects of adaptation, the utility for common chronic illness was unexplored. Other scales include the Psychosocial Adjustment to Illness Survey to assess adaptation in the psychosocial mode and the Psychological Adaptation Scale to observe adaptation in the psychological mode (Biesecker et al., 2013; Kolokotroni et al., 2017) whereas the physiological mode, perception of illness, and environmental factors are insufficient. Overall, to assess adaptation, a reliable, valid, and culturally acceptable assessment tool with a solid theory base is crucial.

The Roy Adaptation Model (RAM) has provided a perspective to understand coping with stimuli, recognizing coping as a multidimensional and transactional process (Roy, 2011). The Roy Adaptation Model is an advanced and widely used conceptual model for nursing that has been evolving since the late 1960s. Roy described humans as an adaptive system with coping processes acting to maintain adaptation in the four adaptive modes, including physiological/physical, self-concept/group-identity, role function, and interdependence (Roy, 2009). Coping processes are delineated by cognator and regulator subsystems. When coping with changing circumstances, the regulator system relates to physical processes, and the cognator involves cognitive and emotional processes. Adaptation is used to portray the integration of human and environmental resources with thinking and feeling and the individuals’ conscious awareness and choice (Pollock, 1986; Roy, 1997). Overall, adaptive modes and coping processes have been adequately explored from a theoretical standpoint.

Roy reviewed over 40 years of RAM-based research (Roy, 2011). At the operational level, various middle-range theories derived from the RAM were developed for the explanation of adaptation in different contexts (Dobratz, 2008). For example, the Middle-Range Theory of Coping and Adaptation Processing (MRT-CAP) has guided an in-depth understanding of coping. It demonstrates that the four adaptive modes, physiologic, self-concept, role function, and interdependence, can be observed through responses and behaviors of the individuals during the coping and adaptation processes (Roy, 2011). As noted in the MRT-CAP, the input, central, and output phases of cognitive processing were described and reflected the coping response (Roy, 2011). Additionally, cognitive processes reflecting patients’ perception of illness and patterning of coping behaviors were also elaborated in the adaptation processes, which are consistent with other studies (Beutel, 1985; Lenzo et al., 2019). Previous studies maintain that adaptation is a complicated and dynamic process and requires ongoing assessment (Barone et al., 2008; Biesecker et al., 2013), however, well-constructed instruments are needed to explore the strategies used by patients in the coping process in order to guide practice.

Although 123 instruments were developed from the RAM, none was able to measure the holism of persons as an adaptive system (Barone et al., 2008). Notably, the 47-item Coping and Adaptation Processing Scale (CAPS) was developed based on the MRT-CAP and related empirical work. The 47-item CAPS included five factors, namely, resourceful and focused, physical and fixed, alter processing, systematic processing, and knowing and relating (Roy, 2011). Each item shows a coping skill that an individual may use to respond to a crisis. Previous studies confirmed the use of the 47-item CAPS as a practical tool to effectively measure coping and adaptation processing in people with chronic and acute health conditions in different countries (Roy, 2011; Alkrisat and Dee, 2014). To enhance and make CAPS appropriate for use in varying cultures, a revised 15-item CAPS based on the MRT-CAP has been created using the item response theory (Roy et al., 2016). According to a study by Roy et al. (2016) the CAPS–Short Form (CAPS-SF) items selected from 47-item CAPS were related to the unidimensional concept and represented across each of the conceptual elements of the MRT-CAP. To date, the English and the Korean versions of the 15-item CAPS have been tested in patients with chronic neurological deficits, cardiac conditions, and cancer (Roy et al., 2016; Song et al., 2018) but further studies on exploring the types and characteristics of inferred coping strategies proposed in the MRT-CAP are encouraged.

Little is known regarding the application of the assumptions and alignment of the RAM and MRT-CAP in the context of the Chinese culture. Thus, empirical verification of the CAPS among the Chinese populations is required. Initially, Yan Tuo shortened the CAPS from 47 items to 28 items and applied it in a cross-sectional study to assess individuals’ adaptive ability (Tuo and Jiang, 2009) but the ongoing assessment of its psychometric properties is lacking. Using the 15-item CAPS-SF to test the theoretical concepts from the MRT-CAP would contribute to the knowledge of coping strategies and adaptive behaviors of the Chinese populations. Hence, the development and validation of a Chinese version of chronic diseases are significant.

As a hypothesis, a greater capacity for using effective coping strategies can improve adaptive behaviors. The Chinese version should measure the individuals’ capacity of coping within adaptive modes. Consequently, this study aimed to develop a psychometrically sound Chinese version of CAPS-SF (hereafter CAPS-SF-C) among patients with chronic illness in mainland China.

## Materials and Methods

### Study Design

A descriptive survey study design for translation, adaptation, and validation of the CAPS-SF in Mainland China was conducted. The study was completed in June 2019. The STROBE (Strengthening the Reporting of Observational studies in Epidemiology) statement was used to report the study.

### Participants

Using a convenience sampling, participants (*n* = 81) with chronic diseases undergoing regular follow-up clinic visits in the targeted hospital were approached to participate in the cultural adaptation of CAPS-SF from May to June in 2018. Eligibility criteria were (1) adults (≥18 years); (2) diagnosed with a specific chronic disease, such as heart failure, diabetes, and hypertension; (3) receiving regular medical treatment; (4) no cognitive impairments; and (5) able to understand the questionnaire and complete in Mandarin. This was followed by the recruitment of a convenience sample of 288 participants for validation of the CAPS-SF-C from January to June in 2019. Taking into consideration participants disease burden, of the 288 participants, 20 participants were randomly selected for a retest; 40 participants with heart failure were randomly selected to complete a Quality of Life measure; and 120 participants were invited to complete an extra coping scale.

### Instruments

#### Demographic Characteristic

An investigator-developed form was used to collect data on the demographic characteristics (age, sex, marital status, religion, education level, job, and family income), medical histories, and treatment histories of the patients.

#### The Coping and Adaptation Processing Scale–Short Form

The 15-item English-language CAPS-SF measures the key concepts of the MRT-CAP and is based on the RAM. In the CAPS-SF, each item is rated on a four-point Likert scale: 1 = “never,” 2 = “rarely,” 3 = “sometimes,” and 4 = “always.” The range of scores is from 15 to 60, with a high score indicating a more consistent use of the identified strategies of coping. The initial psychometric properties of the CAPS-SF were evaluated by internal consistency (Cronbach’s α = 0.82), face validity (theory-based), concurrent validity (correlating with a quality-of-life measure, *r* = 0.38, *p* > 0.05), and divergent validity (correlating with a self-report of cognitive deficits of difficulty in concentration and memory, *r* = −0.39, *p* > 0.05) (Roy et al., 2016).

#### The Medical Coping Modes Questionnaire

In the field of health care, Feifel developed the MCMQ, with three coping styles of confrontation, avoidance, and acceptance–resignation (Feifel et al., 1987). The adapted Chinese version of the MCMQ comprised 20 items with four-point (1–4) Likert scales (Shen and Jiang, 2000) which has been widely used in clinical settings. The MCMQ scores were used for testing concurrent validity.

#### The Minnesota Living With Heart Failure Questionnaire

Health-related quality of life (HRQOL) in physical, functional, social, and emotional domains denotes the individual’s health outcomes and perceived satisfaction when living with chronic diseases (Stanton et al., 2007). Indicators of disease-related symptoms and concerns differ among various HRQOL assessment tools. In this study, the Chinese version of Minnesota Living With Heart Failure Questionnaire (MLWHF), a disease-specific questionnaire for heart failure patients, was selected to measure HRQOL. The 21-item MLWHF is a 6-point Likert scale (0–5) to assess individuals’ quality of life in domains of symptom management and physical and emotional functions. As reported, the Cronbach’s α coefficients of each subscale were 0.88, 0.81, and 0.83 (Zhu et al., 2010). As such, the scores of MLWHF were obtained from patients for evaluating predictive validity.

### Study Procedure

Permission to use instruments was obtained from their authors. Following the recommended procedures for the cross-culturally validated research instruments, translation, adaptation, and validation, the development, and psychometric testing of CAPS-SF-C were conducted (Sousa and Rojjanasrirat, 2011; Streiner and Kottner, 2014).

#### Translation Procedure

The translation procedure comprised four steps (1–4).

***Step 1.*** *Forward translation:* Two authors who were proficient in English and Mandarin translated the CAPS-SF into Chinese independently.

***Step 2.*** *Synthesis of the two translated versions:* After the comparison and integration of items and the response format of the two forward-translated versions, the initial CAPS-SF-C was formed. All agreements, ambiguities, and discrepancies were discussed and resolved by our eight-member research group. The group consisted of three translators, four investigators, and one professor of nursing.

***Step 3.*** *Back-translation:* Two independent translators, both of whom were completely blind to the original CAPS-SF, translated the initial CAPS-SF-C back into English for clarification of words and sentences.

***Step 4.*** *Synthesis of the two back-translated versions:* Our eight-member research group discussed the wording, grammatical structure of sentences, meaning equivalence, and relevance of the two back-translations. Additionally, ambiguities and discrepancies were sent to the translators for clarity. Finally, the integrated version, two back-translation versions, and translation issues were sent to the original author for confirmation.

#### Cultural Adaptation Procedure

The cultural adaptation process comprised an additional two steps (5 and 6).

***Step 5.*** *Evaluation of the conceptual and content equivalence of items for the CAPS-SF:* The Delphi survey experts were consulted, including one mental health specialist, an advanced nursing practitioner, one clinical specialist, one general physician, one senior nursing researcher, and a nursing professor. The Delphi survey included the original English CAPS-SF and its overview (key concepts), the integrated version, and assessment form for content equivalence (using the following scale: 1 = not relevant, 2 = unable to assess relevance, 3 = relevant but needs minor alteration, 4 = very relevant and succinct). Self-reported biographical information (education level, working experience, research areas, and familiarity with the topic of this scale) was sent to these experts in-person and through e-mail. All the experts sent back their comments and assessment results. All comments for further revisions were compared among the eight-member research group and a consensus achieved through the discussion. Finally, the prefinal CAPS-SF-C was generated for testing with patients.

***Step 6***. *Pilot testing:* On average, the questionnaire took approximately 5 min to complete. The first pilot study was conducted among 32 patients after whom revisions were conducted for reconciliation. The second pilot test was conducted among 49 patients using the revised CAPS-SF-C. Participants were invited to provide suggestions and comments on how to rewrite statements for each item to make expressions clearer. Patients were encouraged to express their interpretations of each item in their own words. Qualitative data collected from the participants and investigators were combined with the patients’ quantitative rates on the CAPS-SF-C. The conceptual linkage between theoretical concepts and each item of the CAPS-SF-C was analyzed by our eight-member research group. Moreover, the assumptions of inferred adaptive behaviors were categorized (Table 1). Based on the patients’ responses, literature review, expert panel comments, and investigators’ feedback, the final CAPS-SF-C with its initial factor categories was generated for theory testing and psychometric evaluation.

**TABLE 1:** Conceptual linkage between adaptive modes and information processing for each item of the 15-item Coping and Adaptation Processing Scale within Chinese culture.

Items	Adaptive modes	Information processing	Inferred adaptive behaviors
(1) Can follow a lot of directions at once, even in a crisis.	Self-concept	Central	Factor 1
(2) Call the problem what it is and try to see the whole picture.	Self-concept	Central	Factor 1
(3) Gather as much information as possible to increase my options.	Self-concept	Input	Factor 1
(4) Generally try to make everything work in my favor.	Self-concept	Output	Factor 2
(5) Can think of nothing else, except what’s bothering me.	Physiologic	Input	Factor 3
(6) Try to get more resources to deal with the situation.	Role function and interdependence	Input	Factor 1
(7) Use humor in handling the situation.	Interdependence	Central	Factor 4
(8) Am more effective under stress.	Self-concept	Central	Factor 4
(9) Take strength from spirituality or the success of courageous people.	Self-concept and interdependence	Input	Factor 2
(10) Can benefit from my past experiences for what is happening now.	Self-concept	Central	Factor 2
(11) Try to be creative and come up with new solutions.	Self-concept	Output	Factor 4
(12) Brainstorm as many possible solutions as I can even if they seem far out.	Role-function	Output	Factor 4
(13) Find I become ill.	Physiologic	Input	Factor 3
(14) Too often give up easily.	Physiologic	Input	Factor 3
(15) Develop a plan with a series of actions to deal with the event.	Role function	Output	Factor 4

#### Validation Procedure

Five advanced-practice nurses and four graduate nursing students, who were trained for sample collection, distributed the questionnaires in person. The survey consisted of two parts, the demographic form to collect participants’ characteristics, family income, and medical history, and the CAPS-SF-C. Because participants could regard a stressful event as a crisis, participants were asked to focus only on their chronic illness experiences when answering the questions.

### Data Analysis

According to COSMIN (Consensus based Standards for the selection of health status Measurement Instruments checklist) (Mokkink et al., 2010b), content validity, construct validity, concurrent validity, predictive validity, internal consistency, test–retest reliability, and a floor/ceiling effect of the CAPS-SF-C were tested. Raw scores from the MLWHF were tabulated into standardized 100-point scales as per the scoring manual. Scores for three dimensions of the MCMQ were summed up separately. The summed scores were used within a latent variable framework (McNeish and Wolf, 2020). Further, the scores of item responses from 15-item CAPS-SF were summed up to achieve an approximation of adaptive capacity.

The data analysis was performed using the SPSS version 22.0, AMOS version 22.0, and jamovi version 1.1.9. Two-tailed tests were calculated with a *P* value of 0.05 as the significance level. The descriptive analysis was used to report the mean and standard deviation (SD) of the continuous variables and percentage frequency for the categorical variables.

#### Content Validity

Content validity (including face validity) was evaluated based on the experts’ ratings on the assessment form using the content validity index at the item level (I-CVI) and the scale level (S-CVI). Experts considered the feasibility of the CAPS-SF-C for Chinese adults living with chronic diseases as relevant.

#### Construct Validity

Confirmatory factor analysis (CFA) was conducted to test construct validity. The sample size in this study met the criteria for the recommended sample size for CFA (100–400 is deemed adequate, and 200 is deemed most appropriate) (Hair et al., 2010). The normality of data was observed using P-P plot. As a result, the data points coincided with the theoretical line (the diagonal). The skewness and kurtosis were tested, showing to be close to 0. The calculated *Z* score (skewness) and *Z* score (kurtosis) are expected to be in the range of −1.96 to 1.96 at the significance level of 0.05 and −2.58 to 2.58 at the significance of 0.01 (Ghasemi and Zahediasl, 2012). The maximum likelihood estimation for CFA was performed to assess the model fit. Following the guideline proposed by Hu and Bentler (1999) if standardized root mean square residual (SRMR) is close to 0.08 or below; the root mean square error of approximation (RMSEA) is close to 0.06 or below; and goodness-of-fit index (GFI), comparative fit index (CFI), and Tucker–Lewis index (TLI) values are close to 0.95 or greater, the model can be considered a reasonably good fit. In addition, the critical ratio, standard error, individual item reliability, composite reliability (CR), average variance extracted (AVE), and modification indices (MIs) were selected to evaluate the component fit measures.

#### Criterion Validity

Spearman correlation analysis was performed to examine the correlation between the CAPS-SF-C and the MCMQ to test the concurrent validity. Predictive validity was calculated by the correlation between the CAPS-SF-C and the MLWHF. If the *r* value is 0.45 or higher, tools can be labeled as criterion valid (DeVon et al., 2007).

#### Reliability

To test the homogeneity of the items with the construct being measured, a McDonald’s omega coefficient, which rests on the assumptions of the congeneric model, was calculated. As well, a Spearman–Brown coefficient was estimated to evaluate the internal consistency of the CAPS-SF-C. Internal consistency reliability of 0.70 or higher was considered acceptable (McNeish, 2018). To test the stability of the CAPS-SF-C, the test–retest reliability was assessed by the intraclass correlation coefficient (ICC) using a two-way mixed-effects model and a consistency definition. Based on the 95% confidence interval of the ICC, a value between 0.75 and 0.90 is indicative of good reliability (Koo and Li, 2016).

#### Floor/Ceiling Effect

The floor effects for the total scale were assessed by the percentage of the sample size that achieved the lowest score, and the ceiling effects were determined by the percentage of the respondents that got the highest score. A percentage of less than 15 would be acceptable, indicating no floor and ceiling effects (Terwee et al., 2007).

### Ethics Consideration

This study was approved by the ethics committee of Sir Run Run Shaw Hospital, Zhejiang University School of Medicine (grant no. 20181203-7). Written consent was obtained across the study phases after patients’ protection of their anonymity and confidentiality was guaranteed.

## Results

### Translation and Cultural Adaptation

Four bilingual translators accurately translated the CAPS-SF-C, which was confirmed by our eight-member research group. Additionally, the original author endorsed the back-translated English version as retaining the original meaning. At step 6, 13 patients found it hard to understand item 7, whereas 15 patients were not sure about item 13 during suffering and hospitalization due to illness. To revise the sentences, a discussion was conducted among the eight-member research group. Finally, item 7 was revised as “Use humor in facing and handling the challenges caused by the illness,” and item 13 as “I find I become ill and out of sorts.” With the explanation of spirituality as “finding the meaning and purpose in life and feeling of being supported,” patients showed agreement that there were no difficulties in responding to all items.

As shown in Table 1, the items of the CAPS-SF-C for measuring adaptive modes and information processing were summarized from the patients’ perspectives and based on theories. In constructing the CAPS-SF-C, factor 1 (items 1, 2, 3, and 6), factor 2 (items 4, 9, and 10), factor 3 (items 5, 13, and 14), and factor 4 (items 7, 8, 11, 12, and 15) were categorized for inferred adaptive behaviors. Seven of the items were considered in the self-concept mode, three in physiological mode, two in role function, and three related to interdependence. As for information processing, six of the items were suggested in input stage, five in central processing, and four in the output stage.

### Participants’ Characteristics

Of the 288 patients, 276 completed questionnaires for analysis. The mean age of the patients was 59.14 years (SD = 17.16 years). Most patients were male (62.3%), married (87.3%), Han Chinese (99.6%), non-religious (76.1%), and employed (64.5%) and had no high school education (65.6%); 70.7% of patients had a family income over United States $582 per month, and 92.8% patients had a health insurance. Of the 276 patients, the mean duration of experience with chronic diseases was 8.35 years (SD = 7.85 years). Whereas 60.1% of patients had been diagnosed with a chronic disease, and the remaining 39.9% of patients were living with two or more chronic diseases and comorbidities. Some participants had chronic heart failure (38.4%), diabetes (35.5%), hypertension (32.6%), and cancer (7.2%).

### Validity

#### Content Validity

Using six experts and an averaging calculation method, of the 15 items, the CVI of 12 of 15 items was 1.00, and 0.83 for the remaining 3 items. The S-CVI/AVE was 0.97. According to experts’ views, the CAPS-SF-C reflected the framework of MRT-CAP and had logical consistency with the English version. Moreover, the CAPS-SF-C was understandable and acceptable for measuring coping strategies among the Chinese populations.

#### Construct Validity

The model indices, including χ^2^(*p*) = 155.580, *df* = 84 (*p* < 0.05), normed χ^2^ = 1.852, GFI = 0.932, AGFI = 0.903, CFI = 0.949, TLI = 0.936, RMSEA = 0.056, and SRMR = 0.065, indicated an acceptable fit (Table 2). As for component fit measures, the critical ratio of items was 4.02 to 16.12 (*p* < 0.001), satisfying the threshold of ≥1.96; standard error was 0.05 to 0.45 (*p* < 0.001); individual item reliability was 0.54 to 0.84 except for items 5 and 14 (0.37 and 0.41, respectively), satisfying the criterion of >0.50. The CR was 0.64 to 0.90 satisfying the criterion of >0.60. Average variance extracted was 0.59 to 0.64 with the exception of factor 3 (0.41), satisfying the criterion of >0.50, and the MI was less than 3.84. Therefore, a preliminary four-factor model was achieved.

**TABLE 2:** Confirmatory factor analysis for the CAPS-SF-C.

Items	Estimate	Standard error	Critical ratio	Individual item reliability	CR	AVE
Factor 1: Resourceful and focused					0.86	0.61
Item 1	0.74	0.09	7.86	0.54		
Item 2	1.08	0.10	10.34	0.76		
Item 3	1.13	0.11	10.24	0.73		
Item 6	1.00	—	—	0.69		
Factor 2: Self-initiated and knowing-based					0.81	0.59
Item 4	.84	0.12	7.21	0.59		
Item 9	.93	0.13	7.22	0.63		
Item 10	1.00	—	—	0.70		
Factor 3: Physical and fixed					0.64	0.41
Item 5	0.94	0.23	4.02	0.37		
Item 13	1.98	0.45	4.43	0.84		
Item 14	1.00	—	—	0.41		
Factor 4: Positive and systematic					0.90	0.64
Item 7	0.79	0.07	12.15	0.69		
Item 8	0.90	0.07	13.83	0.76		
Item 11	0.97	0.06	16.12	0.83		
Item 15	0.68	0.05	12.92	0.72		
Item 12	1.00	–	–	0.83		

*The default model fit statistics: χ^2^(p) = 155.580, df = 84 (p < 0.05), Normed χ^2^ = 1.852, GFI = 0.932, AGFI = 0.903, CFI = 0.949, TLI = 0.936, RMSEA = 0.056, SRMR = 0.065.*

#### Criterion Validity

For concurrent validity, the CAPS-SF-C had a positive correlation of 0.46 with the strategy of confrontation and a negative correlation of 0.28 with the strategy of acceptance–resignation measured by the MCMQ in a cross-sectional study of 120 participants with chronic diseases (*p* < 0.01) (Table 3). Factors 1, 2, and 4 were positively associated with confrontation-oriented coping (*p* < 0.01). No significant correlations were found between avoidance mode and CAPS-SF-C subscales (*p* > 0.05). Factors 1, 2, and 3 were negatively correlated to the acceptance–resignation of coping mode (*p* < 0.05). For predictive validity, the CAPS-SF-C has a positive correlation of 0.58 with the quality of life measured by MLWHF among 37 chronic heart failure patients (*p* < 0.01).

**TABLE 3:** Correlations of the CAPS-SF-C subscales and MCMQ subscales.

Subscale	Coping–confrontation	Coping–avoidance	Coping–acceptance–resignation
(1) Resourceful and focused	0.47**	0.10	−0.24**
(2) Self-initiated and knowing-based	0.27**	0.04	−0.19*
(3) Physical and fixed	0.04	0.02	−0.32**
(4) Positive and systematic	0.30**	0.16	−0.13
Total scale	0.46**	0.15	−0.28**

**P < 0.01; *P < 0.05.

### Reliability

The correlations between four factors were assessed using Pearson correlation analysis (Table 4). The McDonald’s omega coefficient of the CAPS-SF-C was 0.82. The subscales ranged from 0.56 to 0.88 (*n* = 276) (Table 4). The split-half reliability was 0.87. For test–retest reliability, 20 patients completed the follow-up assessment 4 weeks later. The value of the ICC is shown in Table 4. These findings indicate that the internal consistency of the CAPS-SF-C is acceptable.

**TABLE 4:** The correlations among four factors and reliability of the CAPS-SF-C.

Subscale	1	2	3	4	McDonald’s omega	ICC [95% confidence interval]
(1) Resourceful and focused	1				0.78	0.72 [0.41, 0.88]
(2) Self-initiated and knowing-based	0.60**	1			0.68	0.85 [0.65, 0.94]
(3) Physical and fixed	−0.07	0.21*	1		0.56	0.67 [0.34, 0.86]
(4) Positive and systematic	0.69**	0.25*	−0.38**	1	0.88	0.83 [0.63, 0.93]
Total scale					0.82	0.87 [0.70, 0.95]

**P < 0.001; *P < 0.05.

### Floor/Ceiling Effect

No participant (0%) achieved the lowest possible score (15), and 0.4% (1/276) achieved the highest (60), demonstrating no floor or ceiling effect was detected.

### Names and Description of Factor

The names and descriptions of each factor were presented, considering the items’ definition, key concepts from MRT-CAP, and the middle-range model of cognitive processing derived from the RAM within the Chinese culture (Table 5).

**TABLE 5:** Names and description of factors in the Chinese CAPS-SF.

Factor	Description	Hypothesis statement
RF: resourceful and focused	Individuals reflecting on available resources in the short term to expand input and strengthen control	Self-perception of social role enables efficient information seeking
SK: self-initiated and knowing-based	Strategies using self and others that focus on input, central, and output processes	Inner capacities and related resources from the environment can achieve complementarity
PF: physical and fixed	Physical reactions and input phase of handling situations	Stimuli lead to different levels of physical maladaptation
IS: positive and systematic	Personal strategies to methodically handle situations in central and output processes	Individuals can develop adaptive behaviors in given situations

## Discussion

The findings of this study demonstrated that the CAPS-SF-C was successfully translated and culturally adapted, with acceptable validity, satisfactory reliability, and no floor/ceiling effect. The resulting four-factor model explained the adaptive capacity in the context of chronic illness within the Chinese culture, although the strengths of this study should be interpreted in the context of the study limitations.

The resourceful and focused coping strategy in CAPS-SF-C is evaluated by the items of following directions, mapping a whole picture of the problem, and acquiring more information. This describes the integration of resources from external environments that is achieved by seeking resources, planning, and taking actions in given situations. The findings were congruous with problem-focused coping strategies (Baker and Berenbaum, 2007), enabling individuals to initiate behaviors in a positive mood and learning to tackle the consequences of the illness (Ambrosio et al., 2015). This finding suggests that health care professionals should provide valuable information on the patients’ illness and its management. They should also inform them on how to get useful resources.

Regarding the self-initiated and knowing-based coping, the items of CAPS-SF-C were self-centered such as in the words “in my favor.” Spirituality was framed within the self-concept mode and suggested that a person existed for a purpose and created value and meaning of life (Dobratz, 2016). Seeking supports from healthcare professionals, family/friends, and peers, which is common in dealing with chronic diseases, is related to others’ resources and the expansion of an individual’s inner strength during adaptation process. These approaches for self-reformation are in line with a previous study that psychological adaptation involves effective coping, self-esteem, social integration, and spiritual/existential meaning (Biesecker et al., 2013). A qualitative study showed that intensive care unit patients would explore how to ignite and maintain the spark of life to improve their inner strength and willpower, and the coping skill of learning from past life experience was a useful method (Alexandersen et al., 2019). Therefore, it is important to focus on activation of the individual as a leader in their illness management.

The physical and fixed pattern reflects physical feedback, which can be a variable to assess the effectiveness of coping (Baker and Berenbaum, 2007). The inclusion of three reversed items (“can think of nothing else, except what’s bothering me,” “find I become ill,” and “too often give up easily”) on factor 3 indicated individuals’ physical reaction and input phase of handling situations. In this study, we found that some participants could not understand the word of “ill” after being diagnosed with an illness. Because of long duration of the illness, patients accepted that they were confronted with disease-related tasks that existed in their daily life. To some extent, the behaviors in this pattern were closely aligned to emotional-focused coping. Neglecting of the situational context by individuals is problematic, even though defense mechanisms are a conceptual aspect of adaptation (Beutel, 1985). Assessing the physiological adaptation of individuals is associated with the exploration of the dysfunctional metacognitive beliefs, which is related to anxiety, depression, and perceived quality of life (Alexandersen et al., 2019; Lenzo et al., 2019).

Positive and systematic coping summarized the skills used for solving the problem. Empirical data showed that the items (“use humor,” “be more effective under stress,” “be creative,” “brainstorm,” and “develop a plan”) in this category were thought to be important coping skills in chronic health condition. These strategies involve showing a “can do” attitude and tendency, looking inside, and finding the role of self when facing a stressful event, which is similar to self-efficacy and confidence (Schulman-Green et al., 2012). In addition, self-management is inevitable and improves the ability of personalized care planning that is important for patients with chronic illness (Bodenheimer, 2002; Schulman-Green et al., 2012; Coulter et al., 2015). Therefore, this pattern could be used to measure the ability of patients to develop coping behaviors.

In this study, patients involved in the investigation progress provided information about their illness perception and the experience of health management to explain the coping behaviors empirically. Through the translation–validation process, four categories of CAPS-SF-C were extracted, which showed the pattern of coping behaviors. Overall, personality, resources, and social support can contribute to a better understanding of adaptation to chronic diseases (Schulman-Green et al., 2012; Biesecker et al., 2013; Adams and Dahdah, 2016; Kristjansdottir et al., 2018). Although the causes of health crisis can be both internal and external, the coping processes involving different adaptive modes and intriguing adaptive/non-effective behavior are important for illness management. This theoretical contribution allows us to understand the knowledge of RAM and MRT-CAP within the Chinese context. To verify the concepts and statements, the identified model should be clarified and confirmed in clinical practices within the given culture.

The CFA with acceptable indices of fitness supported the four-factor structure. The finding underpins the theoretical of the CAPS-SF-C and is consistent with previous studies (Chayaput, 2004; Song et al., 2018). The total scale is adequate to measure adaptive capacity in line with the definition of adaptation. The S-CVI/Ave of 0.97 indicated high agreement among raters, thus showing good content validity. The values of CR and AVE demonstrated that the studied tool had a good convergent validity except for factor 3. The values of square root of AVE of each factor were higher than correlation coefficients among four factors. This indicated that the tool had good discriminant validity. Future studies are required to test the convergent and discriminant validity using other empirical indicators. Regarding the concurrent validity, the CAPS-SF-C had a consistent pattern of the correlations of the confrontation of the MCMQ. Greater ability of adaptation to challenges positively correlated with the coping styles of confrontation but negatively correlated with the acceptance–resignation. However, it was not correlated with avoidance-oriented coping. Avoidance is considered as a passive coping strategy (Feifel et al., 1987). The concept of adaptation tends to identify a positive power to deal with the crisis and challenges (Audulv et al., 2016). The testing of predictive validity based on correlations between the scores of the CAPS-SF-C and MLWHF supported that promoting the adaptive capacity of coping can contribute to improved quality of life in a given context (Bishop, 2005; Leonidou et al., 2019; Livneh, 2015).

The results from the reliability assessment indicated that the CAPS-SF-C was an acceptable instrument. The McDonald’s omega coefficient of a total of the CAPS-SF-C was at 0.82, and the ICC value was at 0.87. As reported, the Cronbach’s α of the English version was 0.82 (Roy et al., 2016) and the Korean version was 0.83 (Song et al., 2018). Notably, factor 3, including three reversed items (item 5, 13, and 14), had weaker internal consistency, especially items 5 and 14. This finding was consistent with another study on the CAPS, which indicates that these items should be improved (Alkrisat and Dee, 2014). Using regular and reversed items on one scale is recommended to reduce response style bias. However, some studies argued that the results should be considered with caution because of the potential effect of language style and less familiarity with negative words (Suarez-Alvarez et al., 2018) especially when applying the mixed-worded scale in a cross-cultural study. Because of the need to describe the physiological adaptive mode, reversed items were retained, but they require further examination.

Some limitations of this study should be considered. First, the findings of this study might not accurately represent the entire Chinese population because we enrolled participants from Han ethnic group at a single tertiary hospital. The coping strategies they adopted might have some differences compared with other populations. Therefore, the interpretation of the results of categorized patterns should be considered with caution. Second, the number of patients with different chronic diseases was not balanced. Therefore, studies with larger sample size collected from multiple centers and with common chronic diseases, such as cardiovascular diseases, metabolic syndrome, and cancer are advocated to evaluate the utility of the CAPS-SF-C. Third, there were no adequate instruments for comparison as a gold standard for evaluating criterion validity. The original longer version of the scale can be considered a gold standard compared to its shorter version (Mokkink et al., 2010a). However, no valid and reliable Chinese version of the 47-item CAPS is available, and little is known about the direct predictors of the outcome of adaptation. Thus, further improvements are required in terms of assessing the correlation between the subscales of CAPS-SF-C and key concepts from the RAM measured by other psychometrically sound instruments.

## Conclusion

The findings of this cross-sectional study identified the Chinese version of CAPS-SF as a reliable and valid scale with good utility. The Chinese CAPS-SF can be a practical tool to evaluate individuals’ capacity for using the four patterns of coping behaviors among Chinese adults with chronic diseases. Health professionals can use it to help patients enhance their coping strategies toward illness management. Further empirical evidence supporting its application is expected from the ongoing assessment of the CAPS-SF-C in Chinese population.

## Data Availability Statement

The datasets generated for this study are available on request to the corresponding author.

## Ethics Statement

The studies involving human participants were reviewed and approved by the Ethics Committee of Sir Run Run Shaw Hospital affiliated with Zhejiang University School of Medicine (NO.20181203-7), in accordance with the accepted provincial standard of ethics in China. The patients or participants provided their written informed consent to participate in this study.

## Author Contributions

XW, LT, and ZY contributed to the conception and study design. XW performed the data analysis and was responsible for drafting and modifying the manuscript. JS, ZY, and DH made critical revisions to the manuscript. ZY and LT provided the administrative support. LT and JS were involved in translation procedure. RQ and QZ collected the data. All authors contributed to the final approve of this manuscript.

## Conflict of Interest

The authors declare that the research was conducted in the absence of any commercial or financial relationships that could be construed as a potential conflict of interest.
